# Role of Heavy Water in the Synthesis and Nanocatalytic Activity of Gold Nanoparticles

**DOI:** 10.1021/acsnanoscienceau.4c00069

**Published:** 2025-01-07

**Authors:** Nathaniel E. Larm, Christopher D. Stachurski, Paul C. Trulove, Xiaonan Tang, Yun Shen, David P. Durkin, Gary A. Baker

**Affiliations:** † Department of Chemistry, United States Naval Academy, Annapolis, Maryland 21402, United States; ‡ Department of Civil and Environmental Engineering, George Washington University, Washington, District of Columbia 20052, United States; § Department of Chemistry, University of Missouri, Columbia, Missouri 65211, United States

**Keywords:** deuterium, heavy water, D_2_O, gold nanoparticle, deuterium isotope effects

## Abstract

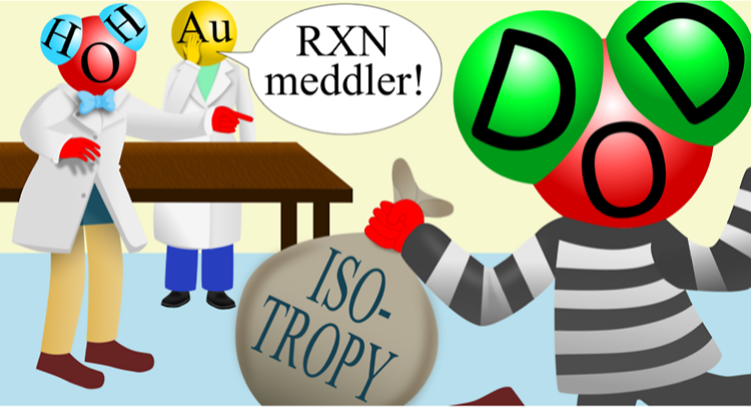

Heavy water (D_2_O) has found extensive application as a moderator in nuclear reactors. Additionally, it serves as a substitute for regular water (H_2_O) in biological or spectroscopic experiments, providing a deuterium source and addressing challenges related to solvent opacity or contrast. This is particularly relevant in experiments involving neutron scattering, infrared absorption, or nuclear magnetic resonance. However, replacing H_2_O with D_2_O is not always a straightforward or harmless substitution and can instead have unintended chemical consequences. In this study, we highlight the significant impact of solvent deuteration on two common gold nanoparticle syntheses—borohydride reduction and ascorbic acid reduction—by comparing reactions in D_2_O and H_2_O and mixtures thereof. The resulting colloids exhibit differences in size and spectral characteristics, and their effectiveness as nanocatalysts in the widely used 4-nitrophenol reduction benchmark reaction is adversely affected by the presence of D_2_O during both particle synthesis and as the catalytic medium. Ultimately, these results underscore a critical awareness often overlooked by scientists and engineers: despite its widespread and sometimes indispensable use in analytical spectroscopy, cellular imaging, biophysics, and organic chemistry, D_2_O cannot truly replace H_2_O without significantly altering the chemical environment of a reaction.

## 1 Introduction

Discrepancies in the physicochemical and biological attributes (e.g., dissociation constant, boiling point, refractive index, and dielectric constant) between standard (light) water (H_2_O) and deuterated (heavy) water (D_2_O) have been recognized for nearly a century. The deuterium isotope effect is a phenomenon associated with differential reactivity or equilibria arising from the effects of isotopically substituting deuterium (D) for hydrogen (H) at one or more places within a molecule. The isotope effect on acid isomerization equilibria, proton transfer reactions, and reaction kinetics (*k*_D_/*k*_H_) in mixtures of light and heavy water has long been known and has offered key insights into acid-catalyzed reaction mechanisms and protolytic equilibria.^1−4^

Within the realm of everyday chemistry, the widespread use of D_2_O in experimental procedures may inadvertently cultivate the belief that it can be interchangeably substituted for H_2_O without significant consequences. For instance, it is routine and standard practice to employ deuteration to attain contrast or solvent transparency in neutron scattering, nuclear magnetic resonance (NMR), and Fourier-transform infrared (FTIR) spectroscopy.^5^ Protein FTIR studies often use buffered D_2_O to shift the bending vibration of water (δ_HOH_) from 1650 cm^–1^ to a lower frequency of 1200 cm^–1^ (δ_DOD_ band). This shift eliminates the troublesome spectral overlap of normal water with the amide I band which carries crucial information regarding protein structure and dynamics.^6^ Of course, this substitution tacitly assumes that biomolecules retain near-native structures and stabilities in solutions partly or wholly comprising D_2_O; it is known that stages requiring biomolecular assembly, activity, and binding can be highly sensitive to H/D substitution. Although replacing H_2_O with D_2_O generally has a limited effect on the biostructure, the differences in hydrogen-bonding ability and the enhanced hydrophobic effect within D_2_O can significantly impact the thermodynamic, structural, dynamical, and ligand-binding properties of proteins and other biopolymers in some cases.^7−9^ Conversely, this provides an elegant and underutilized tool for tailoring hydration strength, enabling precise control and elucidation of the kinetics and intermediates involved in amyloid fibrillization, biomineralization, and protein self-assembly.^10^

In the context of nanomaterial synthesis, the consequences of H/D substitution have been far less explored, understood, or utilized. Bockstaller and co-workers reported that increasing H/D isotopic replacement resulted in systematically higher yields and aspect ratios for gold nanorods prepared following a Ag(I)-mediated seeded-growth method.^11^ Intriguingly, performing the synthesis in D_2_O allowed for an order of magnitude reduction in the concentration of the cetyltrimethylammonium bromide (CTAB) surfactant necessary to facilitate nanorod formation (8 mM vs 80 mM). Gold nanorods prepared in D_2_O with 8 mM CTAB retained uniform particle anisotropy after 1 week, while the same surfactant concentration in regular aqueous solution gave isotropic or cubic particle growth. Puntes et al. investigated the effect of heavy water on the aqueous synthesis of gold nanoparticles (AuNPs) using sodium citrate in an inverse Turkevich method.^12^ They observed a significant reduction in the diameter of AuNPs prepared in D_2_O (5.3 ± 1.1 nm) compared to those in pure H_2_O (9.0 ± 1.2 nm). This difference was attributed to faster reduction facilitated by the higher prevalence of the [AuCl_4_^–^] species, which is the most reactive species in a Turkevich-type reaction. Remarkably, Rodriguez and co-workers recently found that replacing D_2_O for H_2_O under identical hydrothermal conditions selects different thermodynamic products in the synthesis of iron-containing solids, allowing clean access for the first time to phase-pure heterolayered seleno-tochilinite which comprises the layered double hydroxide [Mg_1–*x*_Al_*x*_(OD)_2_]^δ+^ interleaved between (FeSe)^δ−^ layers.^13^ The authors explain this unexpected product selectivity on the basis of the standard reduction potential of D_2_O being 109 mV lower than that of H_2_O at pH 14; i.e., D_2_O is more difficult to reduce. In a similar vein, Zhang and co-workers used deuterated reagents and D_2_O to investigate the reduction pathway of 4-nitrophenol (4-NP) to 4-aminophenol by silver nanoparticles, determining that the amine protons may actually derive from the solvent rather than the reducing agent.^34,35^ Broadly, these studies highlight the potential to further leverage the differences between D_2_O and H_2_O—such as hydrogen bond strength, reduction potential, solvation, and viscosity—to enhance our understanding and control of the nucleation, growth, and evolution of (nano)materials. This approach may also make it possible to access and study materials previously unattainable in normal water.

Here, we extend the extremely limited body of research on D_2_O-based nanoparticle synthesis by investigating the effects of water’s isotopic substitution in two classic AuNP synthesis methods. These methods utilize either ascorbic acid (AA) or sodium borohydride (NaBH_4_) as the sole reducing and capping agent in a simple, ambient, and rapid one-pot preparation. Both syntheses were carried out in solvents with varying degrees of deuteration (reported as estimated atom % D), with the goal of understanding the impact of isotopic substitution on AuNP development, ripening, and catalytic activity. We build on this demonstration by estimating differences in the redox potentials for the agents using cyclic voltammetry (CV), showing that the reducing/stabilizing agent is greatly impacted by the H–D isotope exchange. Finally, we utilize selected AuNPs in a benchmark nanocatalytic reaction—the reduction of 4-NP to 4-aminophenol using borohydride—to demonstrate the effects of D_2_O on both AuNP synthesis and the nanocatalytic conversion process. This study underscores that D_2_O-based solutions provide a significantly different environment compared to their aqueous counterparts while also illustrating how these differences can be leveraged to manipulate nanoscale synthesis effectively.

## 2 Experimental Section

### 2.1 Materials

All experiments involving H_2_O were performed using ultrapure 18.2 MΩ·cm water obtained from a Milli-Q filtration system. Deuterium oxide (D_2_O, Aldrich, 151882, 99.9 atom % D), gold(III) chloride trihydrate (HAuCl_4_·3H_2_O, Aldrich, 520918, ≥99.9% trace metals assay), AA (Fluka, 05878, ≥99.9%), sodium borohydride (NaBH_4_, Aldrich, 480886, 99.9% trace metals basis), potassium chloride (KCl, Aldrich, 208000, 99+%), and 4-NP (Aldrich, 241326, ≥99% assay) were used as received. Notably, while the H_2_O used herein was polished to a purity of 18.2 MΩ·cm in our lab space, we cannot ensure a similar purity for commercial D_2_O. Solvent purity is paramount to ensure reproducibility when synthesizing nanomaterials,^14^ so we conceded to using the commercial D_2_O freshly as received and reserving the bottles for solely this study to prevent contamination.

### 2.2 Characterization

UV–vis spectroscopy was performed using a Jasco V-550 spectrophotometer (400 nm min^–1^ scan rate, 2 nm resolution, PMMA 1 cm path length cuvettes). Transmission electron microscopy (TEM) was performed using an FEI Talos F200X TEM. Samples were dipped on carbon-coated grids purchased from Electron Microscopy Sciences (C-flat holey carbon, 400 mesh, CF-2/2–4C). All electrochemical experiments were conducted under ambient conditions by using a Biologic SP-300 potentiostat. Cyclic voltammograms were measured using a glassy carbon working electrode (Pine Research, 3 mm diameter), a platinum counter electrode, and a Ag/AgCl reference electrode. Working electrodes were polished using a series of alumina slurries (6, 1, and 0.25 μm) prior to initial use and cleaned with a microfiber polishing pad between successive scans.

### 2.3 Synthesis of AuNPs

AuNPs were prepared with a [Au] of 0.25 mM within varying v/v quantities of D_2_O/H_2_O using two established literature methods: sodium borohydride^15,16^ (NaBH_4_; *R* value, or the molar ratio of reducing agent to Au, of 10) or ascorbic acid^17−19^ (AA; *R* value of 3.4) reduction. The exact procedures are provided in the Supporting Information (Table S1). All glassware and stir bars were cleaned with aqua regia prior to use. The v/v quantity of D_2_O is expressed herein as the atom % D in the solvent proper. For example, a 90 atom % D solution comprises a 90:10, v/v mixture of D_2_O and H_2_O. Note that, in this estimation, we consider the H content within the salt precursors (HAuCl_4_·3H_2_O, NaBH_4_, and AA) to be insignificant compared to the H and D provided by the solvent itself. To better illustrate this assumption, consider the molar quantities of hydrogen. In 10 mL of 0.25 mM HAuCl_4_·3H_2_O and 2.5 mM NaBH_4_, the amount of salt-derived H is approximately 0.00012 mol. In contrast, the solvent-derived H in 10 mL of D_2_O (99.9 atom % D, according to the vendor’s information) is essentially 10-fold higher, at around 0.0011 mol. Given that the molar quantity of H from the salt precursors is ∼10% of the residual H in the commercial D_2_O sample, we can reasonably conclude that the salts are negligible sources of hydrogen in these solutions.

### 2.4 CV of Reaction Precursors in Light and Heavy Water

5 mM solutions of HAuCl_4_, AA, and NaBH_4_ were prepared using a stock of 0.1 M KCl in either D_2_O or H_2_O. All electrochemical measurements were initiated from the measured open circuit potential, holding for 30 s before scanning between the two established switching potentials, negative first, based on the determined stability window of each solvent.

### 2.5 Application for 4-NP Reduction

The prepared AA-stabilized AuNP colloids ([Au] = 0.25 mM, with H_2_O or D_2_O as the synthetic medium) were aged for one full day prior to their study as catalysts for the model reduction of 4-NP to 4-aminophenol by NaBH_4_. Initially, 2.10 mL of 0.20 mM aqueous 4-NP and 0.90 mL of 0.10 M aqueous NaBH_4_ (freshly prepared) were combined in a PMMA cuvette, resulting in an intense yellow solution of 4-nitrophenolate (400 nm absorbance). A 0.084 mL aliquot of the AA-AuNPs colloid, prepared in either H_2_O or D_2_O, was added to the cuvette cap. The cuvette was then inverted and mixed for 5 s before being placed into the spectrophotometer. The solution’s absorbance at 400 nm was monitored until the reaction was complete, indicated by a loss of approximately 95% of the initial absorbance (*A*_0_). In this reaction, a 5.0 mol % Au to 4-NP ratio was chosen to be comparable to the mol % catalyst reported in other studies.^19^

Apparent rate of reaction (*k*_app_) and turnover frequency (TOF) values were calculated as analytical metrics for this pseudo-first-order reaction. The *k*_app_ is the slope of the linear portion of a ln(*A*_0_/*A*_t_) versus time plot, where *A*_t_ is the time-dependent absorbance value. TOF is calculated as described previously,^19^ using the molar ratio of Au to 4-NP (5 mol %) and the time required to achieve a ln(*A*_0_/*A*_t_) value of 3 as the reaction time, then correcting for the fact this corresponds to a 95% reaction completion (see Supporting Information for additional details).

## 3 Results and Discussion

### 3.1 D_2_O Slows AuNP Formation via Borohydride Reduction

**Figure 1:**
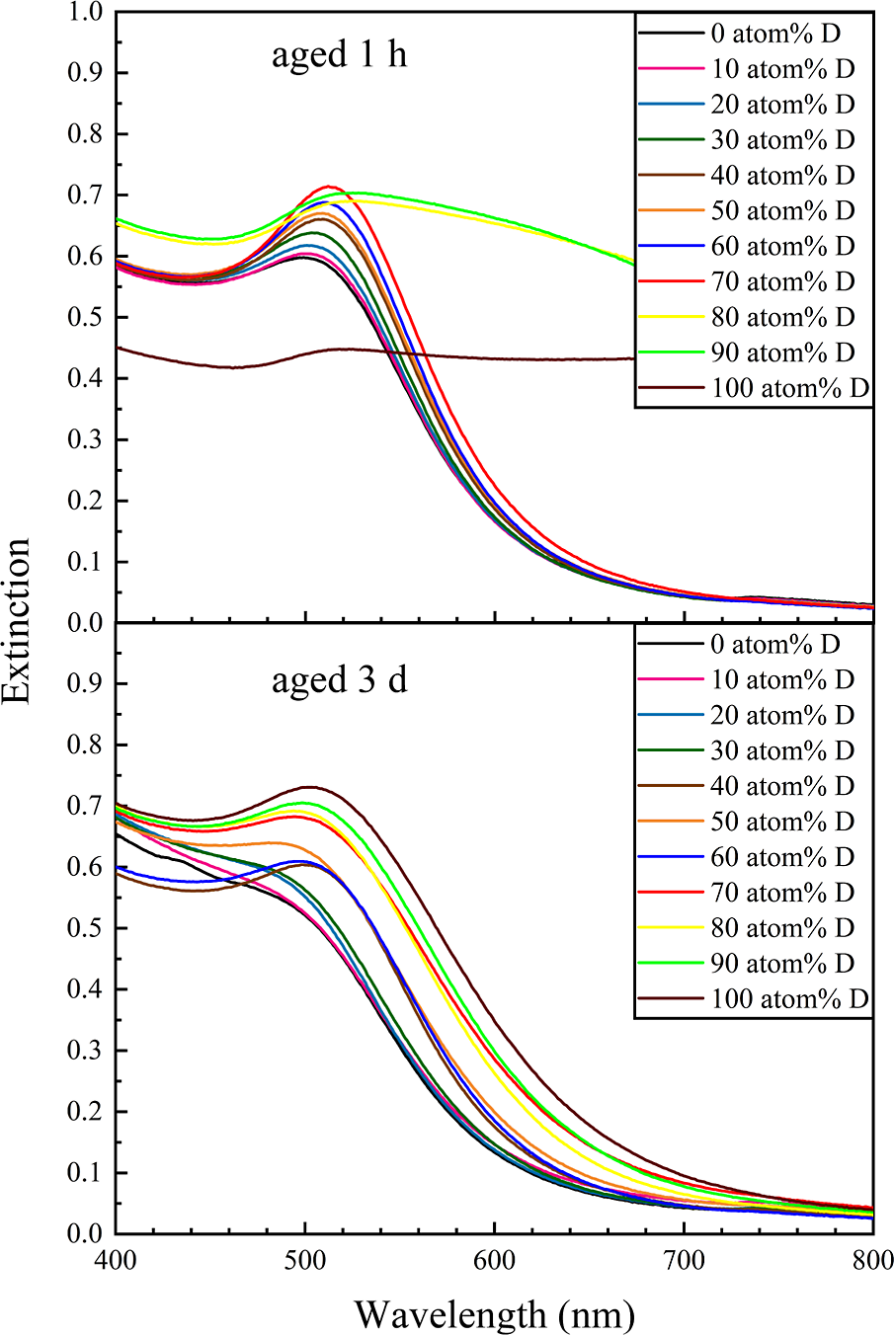
UV–vis spectra of NaBH_4_-stabilized AuNPs synthesized in fully aqueous (0 atom % D) versus increasingly deuterated media (10 to 100 atom % D), measured after aging for (top) ∼1 h and (bottom) 3 days. Significant particle evolution or ripening is observed over time, especially in colloids prepared in a high atom % D aqueous medium.

Borohydride-based AuNP synthesis generally occurs at essentially the speed of mixing, resulting in a nearly immediate color shift from the lemon yellow of the HAuCl_4_ solution to the reddish orange of a sub-5 nm AuNP colloid. In line with this, we observed a rapid reduction for 0–30 atom % D colloids, though colloids for ≥40 atom % D were initially purple, indicating the formation of larger (possibly anisotropic) AuNPs or colloidal assemblies (Figure S1). Colloids prepared in 40–70 atom % D media transitioned to a red orange to red hue within several minutes, with higher atom % D solutions taking longer to develop. Conversely, colloids made in D-rich media (80–100 atom % D) remained purple for several hours before eventually turning reddish orange. This observation is supported by the changes in the localized surface plasmon resonance (LSPR) bands of these colloids, measured after aging for 1 h compared to 3 days, as shown in Figure 1. For the 100 at. % D colloid, we further assessed this growth by monitoring the colloid plasmon band across a 10 h period (Figure S2), noting that the majority of the plasmon narrowing and peak shift from 526 to ca. 510 nm occurs during the first hour of storage. Tentatively, we attribute this phenomenon to slower reduction kinetics. Previous studies have shown that NP synthesis using NaBH_4_ in nonaqueous (or partially aqueous) media can reliably slow NP formation and produce colloidal aggregates. This results in a range of colors, from red to purple, due to the slower degradation of BH_4_^–^ to H_2_ gas.^20−22^ At first glance, we assumed that this could be due to differences in the reduction potentials of the produced HD and D_2_ gases. Indeed, the production of H_2_ as a reducing gas is likely the primary factor driving gold reduction by BH_4_^–^ in water. However, the standard reduction potential of D_2_ is approximately −0.004 V versus the standard hydrogen electrode, and HD has a similar negative potential. This indicates that D_2_ and HD should have only a slightly stronger reducing power than H_2_, suggesting a similar capability for reducing Au^3+^. Alternatively, the pH (or pD) of D_2_O is slightly basic at ca. 7.4, and higher pH values are known to slow the rate of borohydride degradation and concomitant H_2_ production.^22^ Additional considerations include the higher density and viscosity of D_2_O (1.11 g cm^–3^ and 1.25 mPa·s, respectively, at 20 °C) versus H_2_O (1.00 g cm^–3^ and 1.00 mPa·s, respectively, at 20 °C), greater “hydrogen bond” strength, and the increased hydrophobicity of the D atom,^10,23−26^ all of which conspire to promote solvent–solvent interactions and inhibit solvent–solute interactions. Indeed, recent 2D infrared spectroscopic discoveries highlight differences in molecular motions between D_2_O and H_2_O, with stronger intermolecular coupling and more delocalized vibration in H_2_O, possibly contributing toward the generation of H_2_ from BH_4_^–^.^27^ All of these variables affect the mobility of solutes and the evolution or ripening of AuNPs through heavy versus normal water interactions with D_2_O-retarded interactions favoring colloidal assembly or aggregation.

**Figure 2:**
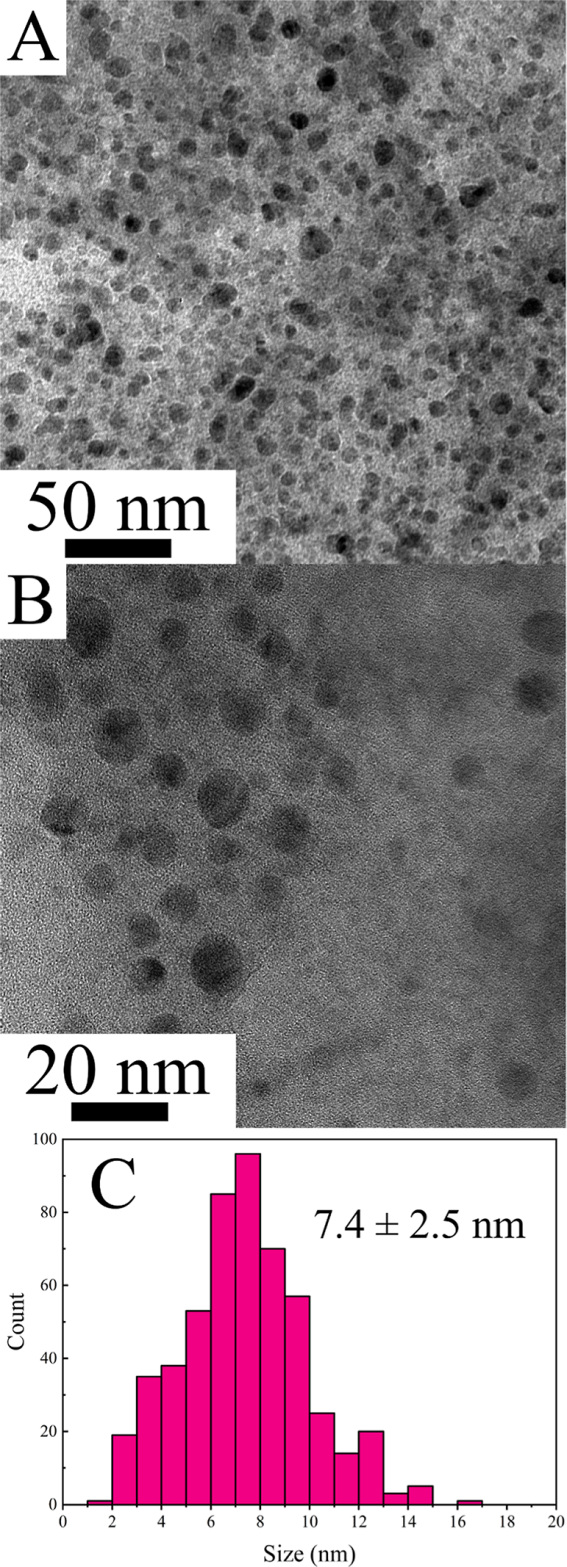
(A,B) TEM images of BH_4_-stabilized AuNP colloids produced in a 100 atom % D environment. Panel C shows the histogram for these AuNPs, with measurements from 436 particles indicating an average diameter of 7.4 ± 2.5 nm. This represents a significant size increase compared to the AuNPs synthesized in 0 atom % D (H_2_O) by Astruc et al., which had an average diameter of ∼2.8 ± 1 nm.^16^

After the generation of reducing gas, freshly reduced Au^0^ atoms likely coalesce into small AuNPs followed by coarsening and aggregation as the free Au^0^ solute is exhausted. This formation mimics the classic Turkevich reaction for citrate-stabilized AuNPs, wherein small AuNPs coalesce into (purple) aggregates prior to fragmentation into smaller discrete particles and the emergence of a distinct red colloid.^28,29^ Imaging by TEM reveals an average AuNP size of 7.4 ± 2.5 nm for BH_4_–AuNPs produced in 100 atom % D solution (Figure 2), including a population of larger particles close to 15 nm in size. This size regime supports the idea that aggregates of smaller AuNPs form a purple colloid before fragmenting over time. Notably, this size significantly contrasts with the 3.1 ± 0.9 nm size observed for similarly prepared 0 atom % D BH_4_–AuNPs (Figure S3) and the 2.8 ± 1 nm size reported by the Astruc group in 2014.^16^ We propose that the slowed kinetics of D_2_O-based AuNP synthesis (due to higher solution viscosity and pH, reduced solvent–solute interactions, and so forth) facilitate the rapid generation of aggregates that coarsen to a final size near 7 nm. Such speculation comports with recent discoveries pointing to 5–7 nm AuNPs as being more energetically favorable with regards to surface energy and stabilizer binding affinity,^30^ a size regime proximal to the AuNPs resulting from NaBH_4_ reduction in D_2_O. In any case, modulating the atom % D in water presents a largely untapped method for controlling the reduction rate of gold and subsequently influencing the size of AuNPs. By adjusting the H/D ratio in water, researchers can, in principle, effectively regulate the reduction kinetics, allowing for more precise control over the formation and growth of AuNPs.

### 3.2 AuNP Shape Control by Ascorbate Isotopologues in D_2_O-Enriched Media

**Figure 3:**
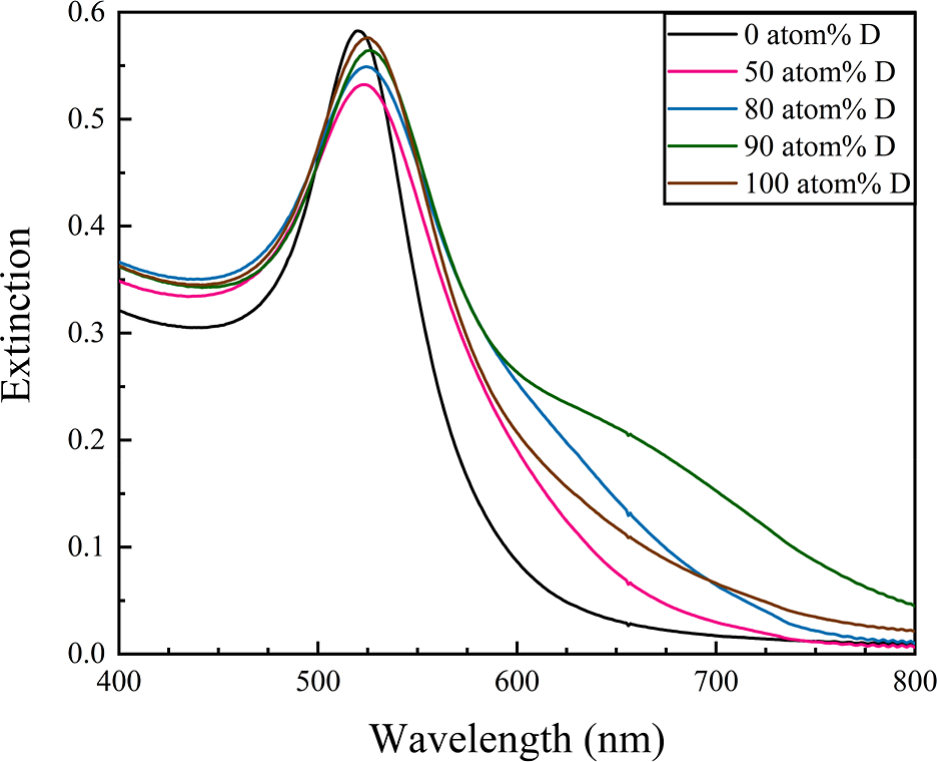
UV–vis spectra showing the LSPR bands of AA-stabilized AuNPs synthesized in normal water (0 atom % D) versus representative deuterated media (50, 80, 90, and 100 atom % D) after aging for 3 days. We note that this sample set was reduced to four deuteration conditions to highlight the impacts of the intermediate versus predominant atom % D while minimizing D_2_O usage.

**Figure 4:**
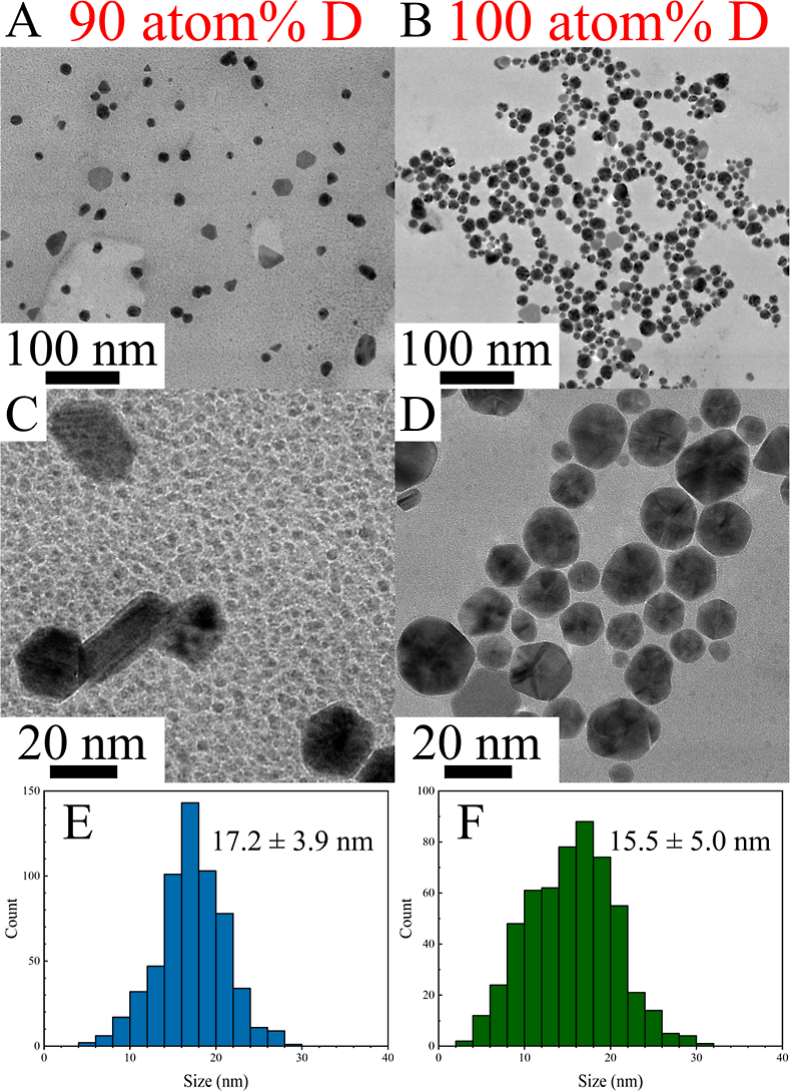
(A,C) TEM images of AA-stabilized AuNPs synthesized in a 90 atom % D environment (H_2_O:D_2_O = 10:90, v/v). Panels B and D depict the corresponding AuNPs prepared in 100 atom % D (pure D_2_O). Panels E and F show the size histograms of the samples made using 90 and 100 atom % D media, respectively (based on >300 AuNP measurements for each). The average AuNP diameters are 17.2 ± 3.9 nm and 15.5 ± 5.0 nm for the 90 and 100 atom % D samples, respectively. This indicates a significant size reduction compared to our previous work in H_2_O which produced AA-AuNPs having an average diameter of ∼30 nm. The broader LSPR bands observed for higher atom % D AuNPs may arise from populations of trigonal and truncated trigonal plates and rods, shapes not observed in an analogous synthesis conducted in H_2_O.

Typical 0 atom % D, AA-stabilized AuNPs using an *R* value of 3.4 possess an average size of 31.8 ± 11.5 nm (Figure S4; in agreement with a prior publication claiming 33.1 ± 9.3 nm with a LSPR peak at ∼525 nm^17^) and are approximately spherically shaped. Interestingly, increasing atom % D to 90% broadens the LSPR band, shifts it red, and evolves a shoulder near 660 nm (Figure 3), indicating larger and more anisotropic NPs. At 100 atom % D, the LSPR shifts slightly blue and loses shoulder definition. TEM images suggest that the quasi-spherical population of AuNPs in 90 and 100 atom % D is smaller in size (17.2 ± 3.9 and 15.5 ± 5.0 nm, respectively) than their aqueous counterpart (Figure 4).^17^ However, there is a significant population of large anisotropic particles (rods, hexagons, and plates) in each colloid, which then contribute substantially to the broadened, red-shifted LSPR band and new shoulder. Likely, the various isotopologues of AA possess different reduction potentials and surface interactions with the resulting AuNPs, such that D_2_O-based colloids are wholly different at the chemical level than their aqueous counterparts. Indeed, previous reports indicate the exchange of ∼4 protons for deuterons in aqueous AA (with a fifth proton being exchanged from the ring C–H)^31,32^ and likely up to 3 in its dehydroascorbic acid oxidation product and further degradants.^33^ This variability in isotopic ligand/reducing agent characteristics results in polydispersity in the final colloid and suggests an exciting opportunity to revisit established nanoparticle syntheses using isotopologue control as a novel synthetic parameter.

### 3.3 Impact of Atom % D on the Redox Potentials Associated with NaBH_4_ and AA

**Figure 5:**
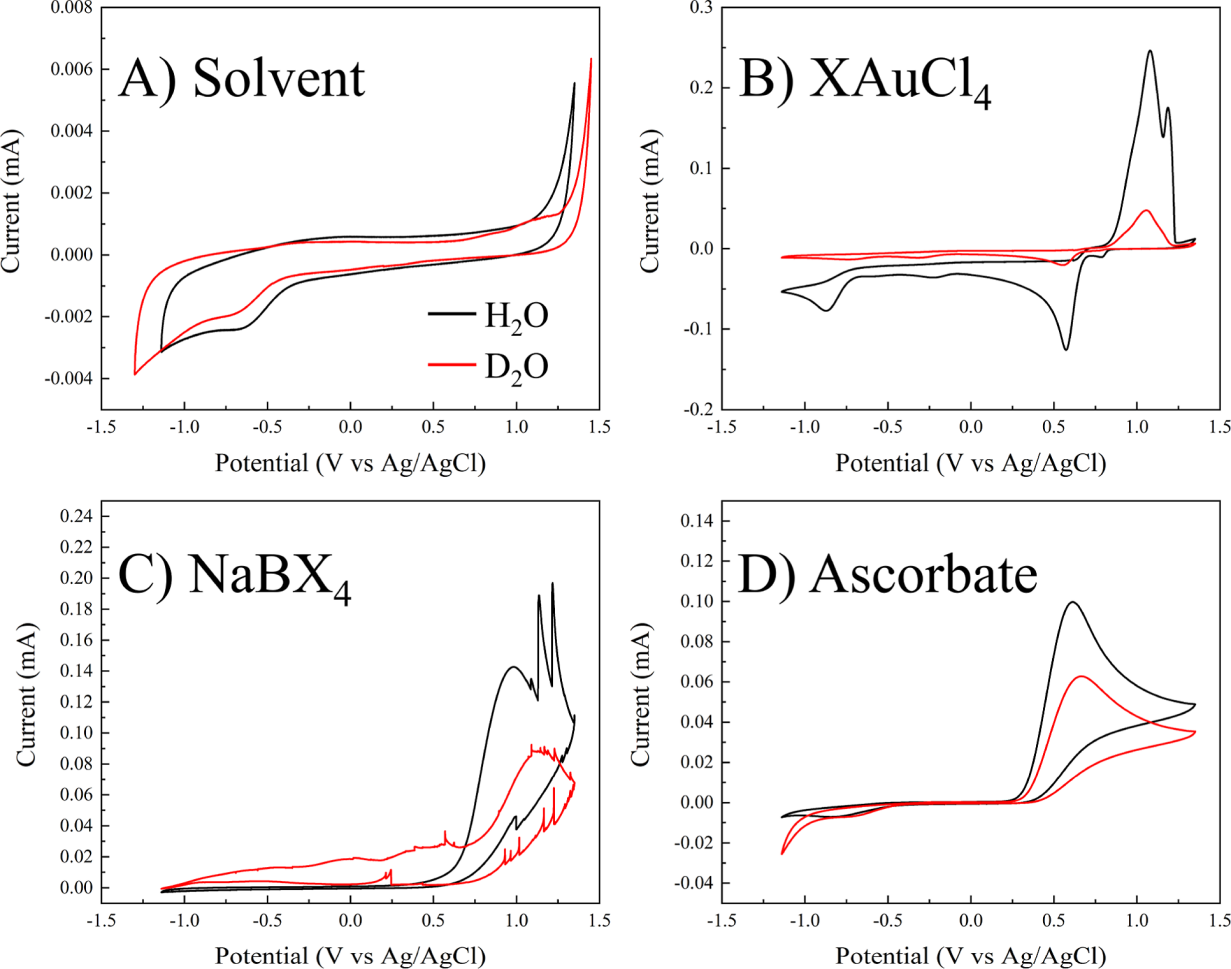
Cyclic voltammograms illustrating the redox potentials of each component in the AuNP synthesis (gold salt, reductant, and solvent) measured in H_2_O versus D_2_O. Panel A displays the neat solvents, panel B shows the gold salt precursor, and panels C and D present the reducing agents (borohydride and ascorbate, respectively). In this figure, “X” denotes either H or D, depending on the H–D exchange. The legend in panel A applies to all panels.

To investigate the impact of atom % D in water on the synthesis of AuNPs, the redox potentials of relevant chemicals in each solvent were measured using CV (Figure 5). A consistent electrolyte (100 mM KCl) was used for each test, made in either heavy or light water, with analytes held at a concentration of 5 mM. We note that due to the low mass of HAuCl_4_ needed to achieve the target concentration and limited material, the actual Au concentration varies slightly between the H_2_O and D_2_O solutions. Most notably, a shift in the onset of oxidation toward more positive potentials can be seen for both NaBH_4_ and AA in D_2_O (Figure 5, panels C and D) signifying weaker reducing agents in the context of AuNP synthesis. In the context of BH_4_–AuNPs, the weaker reducing power could explain slowed reducing gas production and delayed AuNP growth in solutions of increased atom % D, leading to prolonged periods of aggregation. Interestingly, no significant shift in redox behavior was observed for HAuCl_4_ in aqueous water versus heavy water (Figure 5B). This suggests that from a thermodynamic perspective, the behavior of the reducing agent primarily accounts for the observed differences resulting from D_2_O incorporation in the reaction medium. While beyond the scope of this manuscript, one could envision applying a reducing agent isotopologue system where the deuterated form is nonfunctional as a reducing agent. This would allow user control over the formation of metal NPs by dosing protons and pushing H–D exchange.

### 3.4 Slowed Kinetics of AuNP-Catalyzed 4-NP Reduction in Deuterated Media

**Figure 6:**
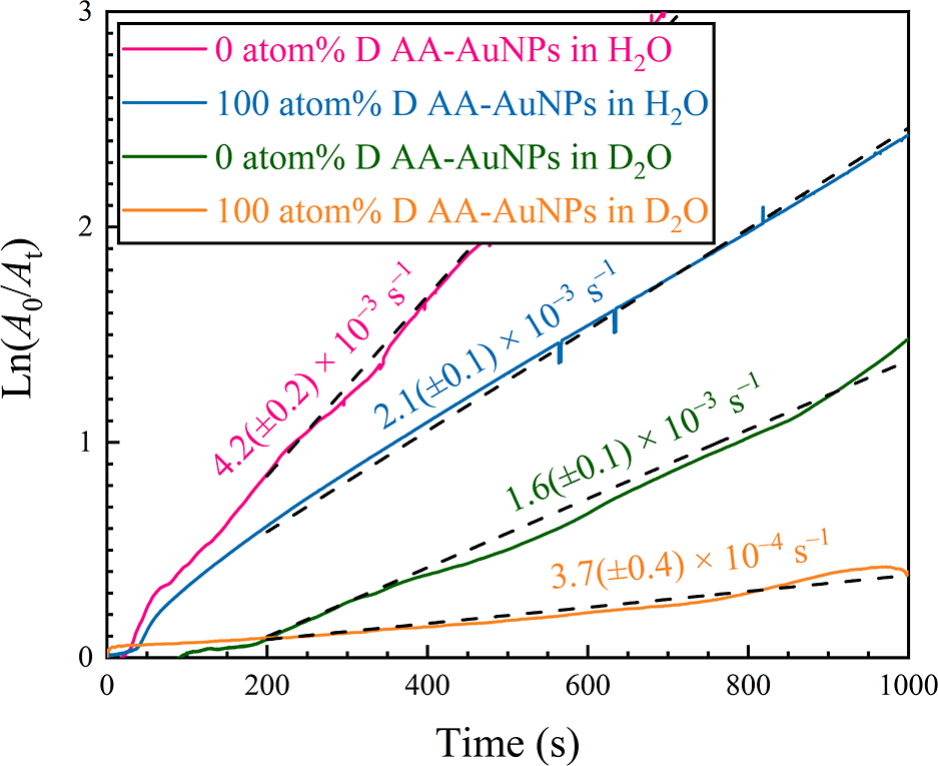
These plots show time-dependent ln(*A*_0_/*A*_t_) results for 4-NP reduction catalyzed by AA-AuNPs synthesized in 0 and 100 atom % D solutions, respectively, labeled “H_2_O” and “D_2_O.” The fastest reaction occurs with AA-AuNPs made in H_2_O and also deployed for 4-NP reduction in H_2_O. The intermediate–activity plot indicates that AA-AuNPs made in D_2_O exhibit half the activity of those made in H_2_O when catalysis is carried out in H_2_O. Even slower kinetics for 4-NP reduction are observed using AA-AuNPs tested in D_2_O as the nanocatalytic medium. This study clearly demonstrates how the deuteration of both the synthetic and the catalytic medium negatively impacts the resulting activity of AuNPs in this benchmark nanocatalytic reaction.

Deuterium incorporation is particularly advantageous for studying nanocatalysts using H-sensitive spectroscopic techniques such as neutron scattering, IR, or NMR. However, synthesizing AuNPs in D_2_O (or D_2_O-enriched media) can significantly impact the performance of the resulting colloids. For instance, when using H_2_O as the catalytic medium, AA-AuNPs prepared in D_2_O display only half of the activity for nitroarene reduction as their counterpart made in H_2_O at the same catalyst loading. Indeed, the apparent catalytic rates (*k*_app_) for 4-NP reduction in H_2_O were 4.2 (±0.2) × 10^–3^ s^–1^ for AA-AuNPs prepared in H_2_O and 2.1 (±0.1) × 10^–3^ s^–1^ for those prepared in D_2_O (TOF values of 98 and 55 h^–1^, respectively; Figure 6 and Table S2). Slowed kinetics have been observed previously for this deuterated reaction,^34,35^ and we tentatively attribute this decrement to the population of larger, somewhat anisotropic AuNPs formed in D_2_O and, possibly, the more hydrophobic nature of the deuterated capping agent isotopologues. Of course, the latter effect persists only until the H–D exchange reduces the population of deuterated AA. When comparing H_2_O- and D_2_O-derived colloids, it is essential to account for differences in AuNP size. In this case, the roughly 2-fold smaller average size of the D_2_O-produced AA-AuNPs suggests they should provide significantly more catalytic surface area compared to the corresponding AuNPs made in H_2_O, given the catalyst loading (5.0 mol % Au) is the same in both experiments. For completeness, we additionally performed the reduction reaction in D_2_O using AA-AuNPs prepared in H_2_O and D_2_O. These both gave even poorer performances, with *k*_app_ values of only 1.6 (±0.1) × 10^–3^ s^–1^ (TOF = 51 h^–1^) and 3.7 (±0.4) × 10^–4^ s^–1^ (TOF = 8 h^–1^), respectively. The significantly slower reaction rates in D_2_O, using the same parent nanocatalysts, underscore the significant differences and negative impact that solvent deuteration has on the performance of this well-studied catalytic reaction. Due to the higher viscosity, basicity, and hydrophobicity of D_2_O (among other factors), H_2_O and D_2_O are clearly not interchangeable as solvents for AuNP synthesis nor as a medium for nanocatalytic reactions.

## 4 Conclusions

The current results support the assertion that heavy water is not interchangeable with light water for nanomaterials synthesis. The distinct characteristics of D_2_O, including its density, viscosity, pH, hydrophobicity, and intermolecular dynamics, make it a fundamentally different solvent from H_2_O. These differences, however, present underutilized yet sophisticated opportunities for controlling nanoparticle synthesis. D_2_O slows the production of AuNPs by borohydride, resulting in larger aggregates and discrete particles compared to those in the aqueous system (7.1 versus 2.8 nm, respectively). Conversely, isotopologues of AA act as competing ligands in D_2_O, decreasing the average particle size while offering potential pathways for introducing anisotropic growth. Furthermore, using AA-based AuNPs as nanocatalysts for model nitroarene reduction demonstrates that the catalytic rate and TOF are significantly reduced in D_2_O compared to that in H_2_O. These findings underscore the significant impact of deuterium in nanoparticle synthesis and function, highlighting its noninnocence in these processes. However, they also suggest that isotopic control can be a valuable and underexplored tool for fine-tuning and customizing nanoscale materials. Ongoing efforts in our laboratories aim to exploit this strategy as a general approach to achieve precise control over nanoparticle shape and function.

## Supporting Information Available

Synthesis and catalysis information, colloid photographs, UV–vis spectra, and TEM images (PDF)
