# Supplementary material for: Role of Heavy Water in the Synthesis and Nanocatalytic Activity of Gold Nanoparticles
Source: ACS Nanosci Au. 2025 Jan 7;5(1):52–9. doi: 10.1021/acsnanoscienceau.4c00069 (PMC11843497; doi:10.1021/acsnanoscienceau.4c00069)
Supplement: Supplementary file 1 — ng4c00069_si_001.pdf [file ng4c00069_si_001.pdf]

SUPPORTING INFORMATION

The Role of Heavy Water in the Synthesis and Nanocatalytic Activity of Gold Nanoparticles

Nathaniel E. Larm,*,† Christopher D. Stachurski,† Paul C. Trulove,† Xiaonan Tang,‡ Yun Shen,‡ David P. Durkin,† and Gary A. Baker*,§

† Department of Chemistry, United States Naval Academy, Annapolis, MD 21402, United States

‡ Department of Civil and Environmental Engineering, George Washington University, Washington, DC 20052, United States

§ Department of Chemistry, University of Missouri, Columbia, MO 65211, United States

*Email: larm@usna.edu; bakergar@missouri.edu

Synthesis of AuNPs using $NaBH_4$. 9.0 mL of H_2O was added to a 20-mL glass scintillation vial equipped with a Teflon-coated stir bar. Next, 0.5 mL of aqueous 5.0 mM $HAuCl_4 \cdot 3H_2O$ was added, and the resulting yellow solution was stirred for 1 min. Finally, 0.5 mL of freshly prepared aqueous 50.0 mM $NaBH_4$ solution was rapidly added while vigorously stirring, resulting in an immediate color shift to reddish-orange. This solution was stirred for 5 min, then the stir bar was removed and the solution was stored in a lab drawer until use. This solution was labeled as the 0 atom% D $NaBH_4$ colloid. Adjustments to the synthesis were performed per Table S1 to achieve a desired final atom% D. Namely, the desired volumetric ratio of H_2O/D_2O was incorporated to achieve atom% D values ranging from 10–90 atom%, and D_2O-based solutions of $HAuCl_4 \cdot 3H_2O$ and $NaBH_4$ were employed for the 100 atom% D colloid. Note that the 100 atom% D solution still comprises approximately 0.11 atom% H (from a combination of solvent contamination and protons present in the gold salt precursor) by rough estimation.

Synthesis of AuNPs using AA. 9.0 mL of H_2O was added to a 20-mL glass scintillation vial equipped with a Teflon-coated stir bar. Next, 0.5 mL of aqueous 5.0 mM $HAuCl_4 \cdot 3H_2O$ was added, and the resulting yellow solution was stirred for 1 min. Finally, 0.5 mL of freshly prepared aqueous 17.0 mM AA solution was rapidly added while vigorously stirring, resulting in an immediate color shift to reddish-orange. This solution was stirred for 5 min, then the stir bar was removed and the solution was stored in a lab drawer until use. This solution was labeled as the 0 atom% D AA colloid. Adjustments to the synthesis were performed as above (see Table S1) to achieve a desired final atom% D.

Calculating apparent catalytic rate and turnover frequency. The apparent catalytic rate (k_{app}) was determined by the linear correlation of the pseudo first-order reduction kinetics for a plot of $\ln(A_0/A_t)$ vs. time. Similarly, the time required for this plot to reach a $\ln(A_0/A_t)$ value of 3 was used to calculate the reaction turnover frequency (TOF). Moles of 4-NP were divided by the product of moles of Au and the required reaction time, correcting for the reaction completion percentage (95% at the aforementioned $\ln(A_0/A_t)$ value of 3).[1]

Table S1. Sample preparation for $NaBH_4$-AuNPs colloids with varying atom% D. RA denotes reducing agent, and is either $NaBH_4$ or AA.

desired atom% D	H_2O (mL)	D_2O (mL)	H_2O $HAuCl_4$ stock (mL)	D_2O $HAuCl_4$ stock (mL)	H_2O RA stock (mL)	D_2O RA stock (mL)
0	9.0	0.0	0.5	0.0	0.5	0.0
10	8.0	1.0	0.5	0.0	0.5	0.0
20	7.0	2.0	0.5	0.0	0.5	0.0
30	6.0	3.0	0.5	0.0	0.5	0.0
40	5.0	4.0	0.5	0.0	0.5	0.0
50	4.0	5.0	0.5	0.0	0.5	0.0
60	3.0	6.0	0.5	0.0	0.5	0.0
70	2.0	7.0	0.5	0.0	0.5	0.0
80	1.0	8.0	0.5	0.0	0.5	0.0
90	0.0	9.0	0.5	0.0	0.5	0.0
100	0.0	9.0	0.0	0.5	0.0	0.5

Table S2. Comparison of 4-NP catalytic effectiveness between AA-AuNPs produced in 100 atom% D or 0 atom% D solutions (5 mol% Au, relative to 4-NP). The solvent heading denotes the reduction medium for 4-NP. The t_{rxn} heading indicates the time required to achieve a $\ln(A_0/A_t)$ value of 3, or 95% reaction completion, as per our published recommendations for reporting on this benchmark nanocatalytic reaction.[1]

colloid	solvent	k_{app} ($\times 10^{-3}$ s^{-1})	t_{rxn} (h)	TOF (h^{-1})
0 atom% D	H_2O	4.2 ± 0.2	0.19	98
100 atom% D	H_2O	2.1 ± 0.1	0.35	55
0 atom% D	D_2O	1.6 ± 0.1	0.37	51
100 atom% D	D_2O	0.37 ± 0.04	2.25	8

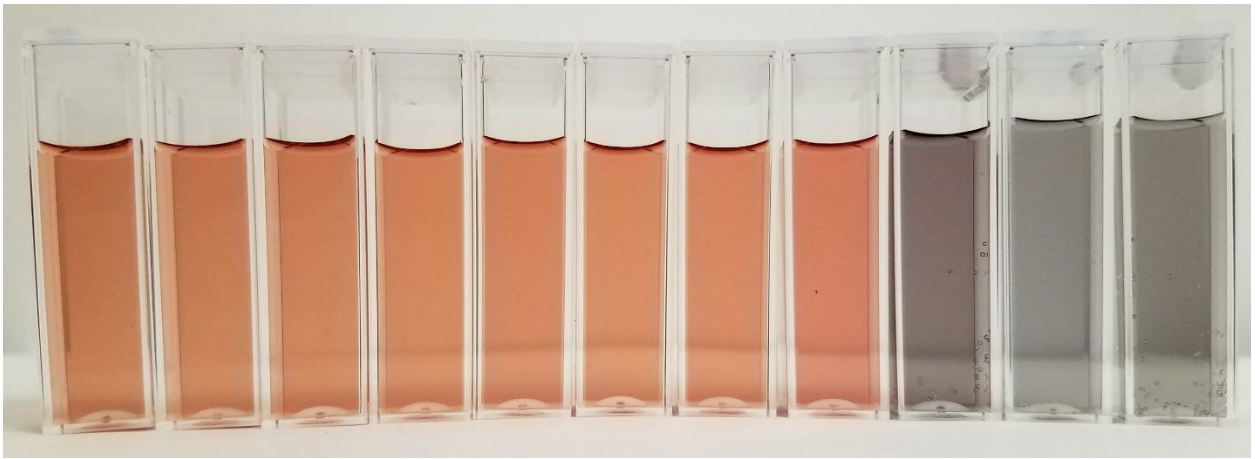

Figure S1. Photograph of AuNP samples prepared by $NaBH_4$ reduction within varying atom% D solutions (left to right, 0, 10, 20, 30, 40, 50, 60, 70, 80, 90, and 100% D_2O) after aging for 1 h. Notably, samples containing 40 to 70 atom% D turned purple immediately upon mixing and then gradually changed to the coral color shown within a few minutes. Samples made in ≥80 atom% D solution became red within 3 d of aging.

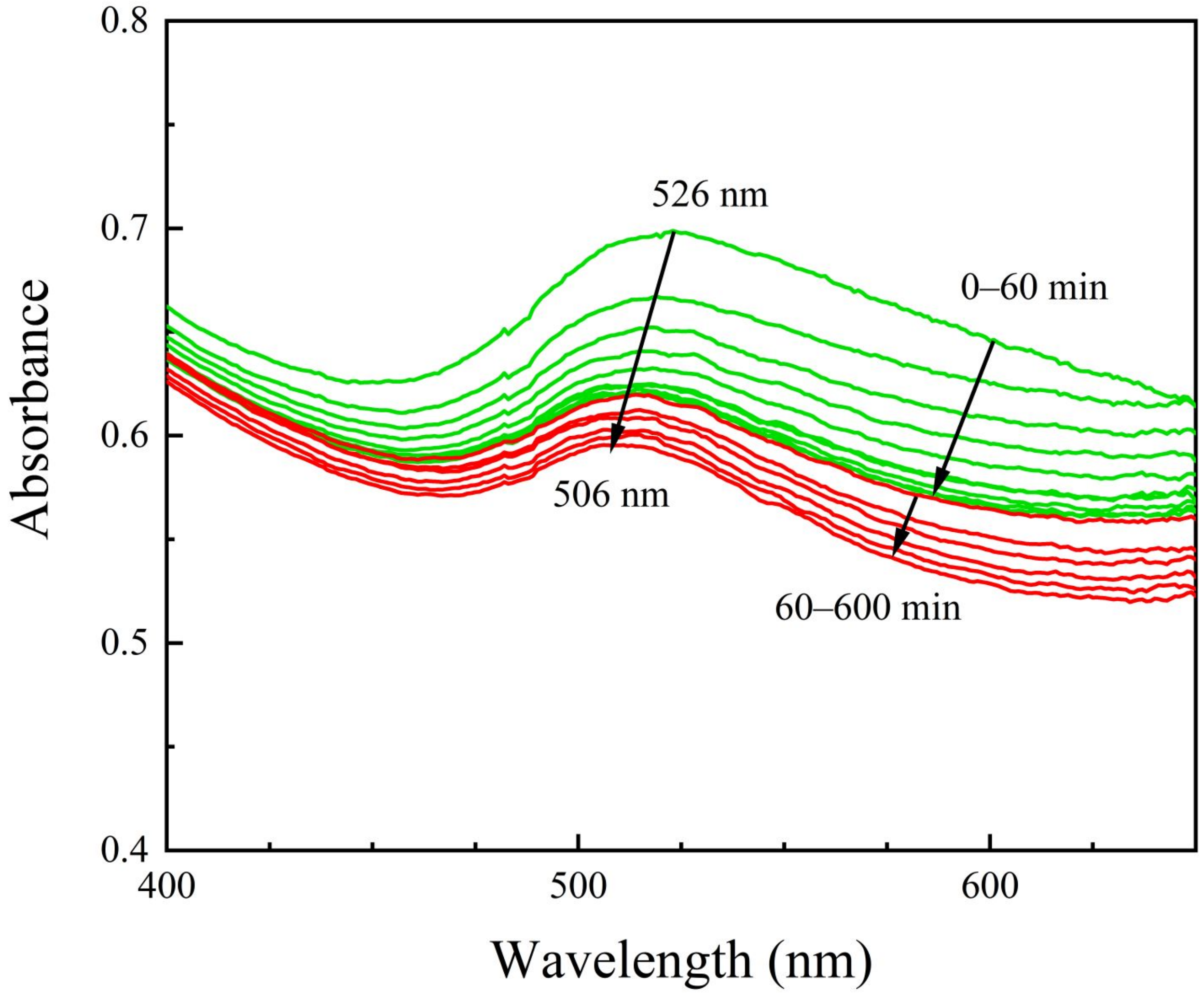

Figure S2. UV–vis spectra depicting the shift in plasmon band for a 100 atom% D colloid of $NaBH_4$-AuNPs stored in a quartz cuvette. During the first hour, the plasmon band narrows slightly and the peak shifts from 526 nm to ca. 510 nm. Over the next nine hours, this trend continues to achieve a peak at 506 nm. Note that this solution remained slightly purple instead of the reddish orange observed for the same colloid stored in a glass scintillation vial. It is possible that storage in quartz coupled with constant monitoring (scans every 10 min) impacted the NP growth.

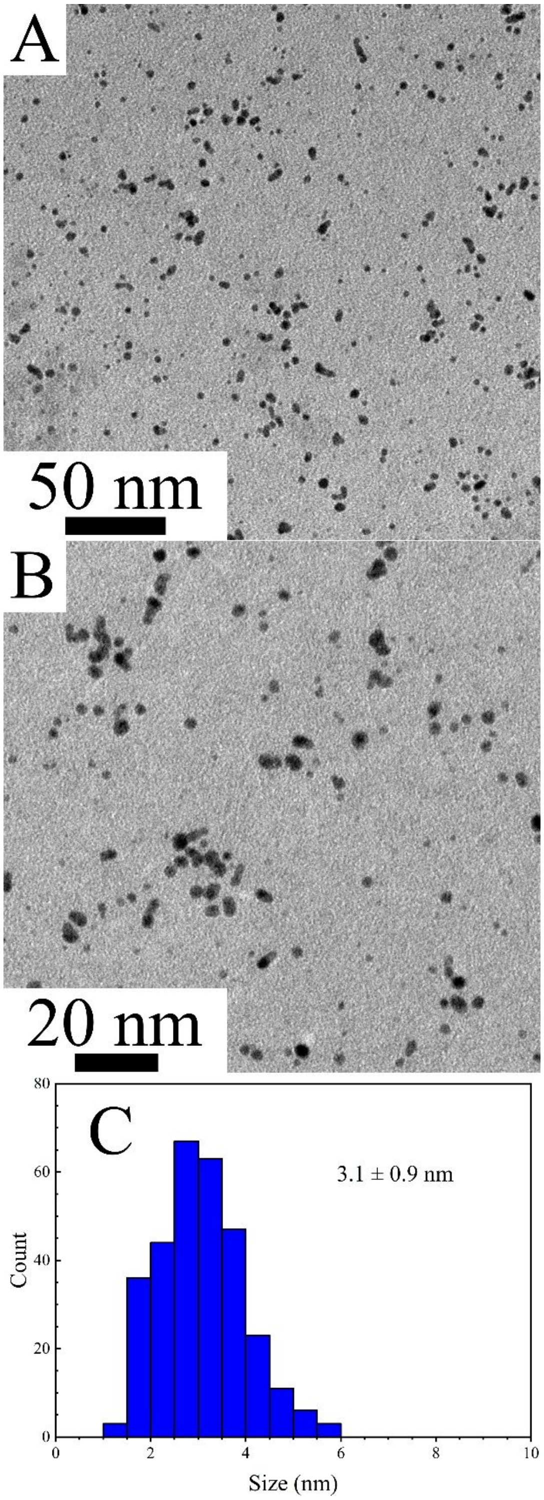

Figure S3. (A and B) TEM images of BH_4-stabilized AuNPs synthesized in an aqueous environment (0 atom% D). Panel C shows the size histogram of the sample (based on >300 AuNPs measured), with an average AuNP diameter of 3.1 ± 0.9 nm. This size regime agrees with prior reports.[2]

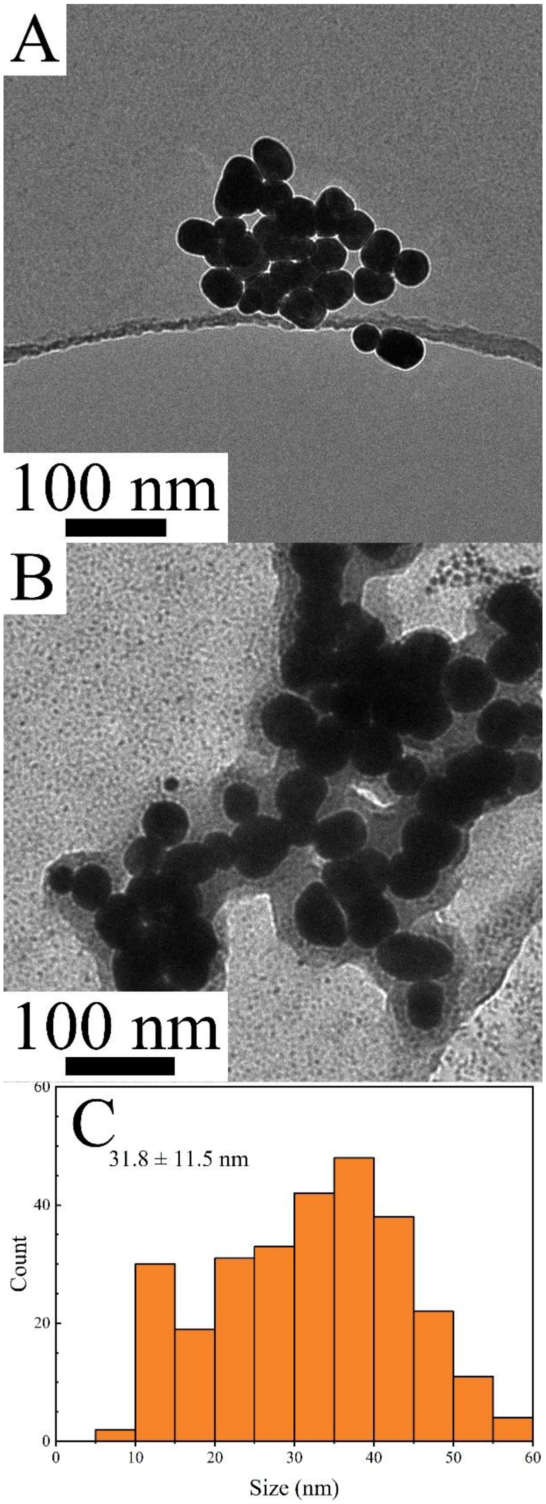

Figure S4. (A and B) TEM images of AA-stabilized AuNPs synthesized in an aqueous environment (0 atom% D). Panel C shows the size histogram of the sample (based on >150 AuNPs measured), with an average AuNP diameter of 31.8 ± 11.5 nm. This size regime agrees with prior reports.[1, 3]

Supporting References

(1) Larm, N. E.; Bhawawet, N.; Thon, J. A.; Baker, G. A. Best practices for reporting nanocatalytic performance: lessons learned from nitroarene reduction as a model reaction. *New Journal of Chemistry* **2019**, *43* (46), 17932-17936, 10.1039/C9NJ01745C. DOI: 10.1039/C9NJ01745C.
(2) Deraedt, C.; Salmon, L.; Gatard, S.; Ciganda, R.; Hernandez, R.; Ruiz, J.; Astruc, D. Sodium borohydride stabilizes very active gold nanoparticle catalysts. *Chemical Communications* **2014**, *50* (91), 14194-14196, 10.1039/C4CC05946H. DOI: 10.1039/C4CC05946H.
(3) Larm, N. E.; Essner, J. B.; Pokpas, K.; Canon, J. A.; Jahed, N.; Iwuoha, E. I.; Baker, G. A. Room-Temperature Turkevich Method: Formation of Gold Nanoparticles at the Speed of Mixing Using Cyclic Oxocarbon Reducing Agents. *The Journal of Physical Chemistry C* **2018**, *122* (9), 5105-5118. DOI: 10.1021/acs.jpcc.7b10536.
